# Creative thinking does not promote dishonesty

**DOI:** 10.1098/rsos.230879

**Published:** 2023-12-06

**Authors:** Moritz Reis, Roland Pfister, Wilfried Kunde, Anna Foerster

**Affiliations:** ^1^ Department of Psychology (III), University of Würzburg, 97070 Würzburg, Germany; ^2^ General Psychology, Trier University, 54286 Trier, Germany

**Keywords:** dishonesty, creativity, thinking style, unethical behaviour, morality

## Abstract

We assessed the relation of creativity and unethical behaviour by manipulating the thinking style of participants (*N* = 450 adults) and measuring the impact of this manipulation on the prevalence of dishonest behaviour. Participants performed one of three inducer tasks: the alternative uses task to promote divergent thinking, the remote associates task to promote convergent thinking, or a simple classification task for rule-based thinking. Before and after this manipulation, participants conducted the mind game as a straightforward measure of dishonesty. Dishonest behaviour increased from before to after the intervention, but we found no credible evidence that this increase differed between induced mindsets. Exploratory analyses did not support any relation of trait creativity and dishonesty either. We conclude that the influence of creative thinking on unethical behaviour seems to be more ambiguous than assumed in earlier research or might be restricted to specific populations or contexts.

## 1. Introduction

Creative thinking fascinates scientists and laypeople alike. Defined by the production of ideas which are novel and useful [1], it is not only the basis of scientific breakthroughs [2], but also plays a major role in everyday behaviour [3]. Accordingly, creativity is commonly seen as socially desirable [4,5] and most people associate creativity with favourable characteristics like high intelligence [6,7].^1^ Despite this inherently positive connotation and decisive calls for more original behaviour (e.g. [10,11]), it has been proposed that there might also be a negative side of creativity [5,12].

At times, high creativity indeed may be used to maximize own profit at the expense of others, a phenomenon referred to as negative or malevolent creativity [13,14]. Criminals, for instance, often display an impressive (and at the same time frightening) amount of creativity when planning and carrying out elaborated robberies. Creativity thus enables certain forms of deviant behaviour because it allows for envisioning sophisticated strategies of how to put a criminal intent into action [15–17].

However, there might not only be ways to act creatively in finding ways to harm others, but creative thinking itself might promote unethical behaviour [18]. This claim is rooted in findings that creativity supports the generation of self-serving justifications for planned dishonest behaviour, so that the agent maintains a positive self-image despite engaging in such activities [19]. In line with such reasoning, dishonest behaviour was associated with trait creativity and it appeared to be promoted by a creative mindset in a series of five experiments [18]. A later study seemed to corroborate the assumed link of creativity and dishonesty [20]. That study, however, later was identified as reporting fraudulent results [21] and has therefore been retracted recently. Moreover, several studies from other research fields which used a similar mindset manipulation to Gino & Ariely [18], i.e. the scrambled sentence test, did not replicate the original finding (e.g. [22,23]).

Other research suggests instead that creativity and *ethical* rather than unethical behaviour draw on similar mental skills, like anticipating the consequences of one's own behaviour [24], which would suggest a positive relation of ethical behaviour and creativity [25]. Adding to the mixed findings reviewed above, a recent correlational study found that results critically depend on the measure of creativity (e.g. objective or subjective creativity) and the operationalization of unethical behaviour [26]. Even though correlational studies are the tool of choice to analyse the relation of different personality traits, they bear the risk of being confounded by unconsidered variables, and leave open the casual direction of possible correlational links (e.g. [27]).

We thus created an experimental setup to systematically test the causal influence of creative compared to rule-based thinking styles on unethical behaviour and therefore shed light on the inconclusive database at hand. Such thinking styles are commonly defined as cognitive states, which regulate attention, thought and behaviour in a particular way [28,29]. Taking the complexity of creative cognition into account, there were two creative thinking groups. These two groups covered the most important facets of creative cognition: divergent and convergent thinking. Even though both of these thinking styles are vital for creativity [30], divergent and convergent thinking often showed contrary effects on human behaviour, e.g. regarding interpersonal trust [29] and affective states [31]. We, therefore, planned to compare both types of creative mindsets to a rule-based control condition while also contrasting both components of creative thinking in their impact on dishonest behaviour. The participants of the control group performed a simple classification task to create a rule-based thinking style, i.e. a cognitive state which is tuned towards rule-abiding behaviour. To instill a primarily divergent mode of thinking, we employed the *alternative uses task* [32]. Finally, to instill a primarily convergent mode of thinking, we used the *remote associates task* [33]. Even though both creativity tasks do not exclusively promote divergent or convergent thinking [34], they differ substantially regarding the predominantly stimulated thinking style.

Before and after these manipulations of thinking style, participants took part in an adapted version of the *mind game* [35,36], as a straightforward measure of unethical behaviour. Participants simply reported whether they came up with the same or a different number as presented on screen, whereby only same reports earned them a monetary bonus, thus promoting untruthful reports. We specifically chose this paradigm as its act of lying does not require any creativity itself, allowing us to capture whether creativity promotes unethical behaviour *per se* instead of coming up with a creative dishonest solution. Following earlier experimental investigations [18], we expected that creative thinking (divergent and convergent thinking) leads to more dishonesty than a rule-based thinking style. Moreover, as divergent and convergent thinking were found to have different effects on decision-making elsewhere (e.g. [29]), we hypothesized that they would also differ in their influence on dishonesty.

## 2. Method

### 2.1. Participants

Our main analysis was a logistic regression with multiple predictors (categorical and continuous), including indicator and difference contrasts. To the best of our knowledge, there is no established way to conduct a power analysis for such an analysis, so that we decided to test twice as many participants as had been used in earlier studies with a comparable study design (150 per group; 450 in total; age: *M* = 27.8, s.d. = 9.8; 179 female, 261 male, 9 non-binary, 1 prefer not to say; cf. 75 participants per group for [37]). As preregistered (https://osf.io/69huj), we excluded datasets of participants with low performance in the thinking style tasks, because the manipulation might not have been successful in this case (see Data treatment and analyses).^2^ Datasets which had to be excluded were replaced by new ones until we reached the planned group size. We therefore collected 537 datasets in total, excluding 87 participants (divergent thinking: 19; convergent thinking: 63; rule-based thinking: 5). We recruited our sample via Prolific and participants reported 42 different nationalities, among which Poland (*n* = 95), Portugal (*n* = 76) and South Africa (*n* = 48) were mentioned most frequently. Participants received £3 for participation as well as the bonus payment they earned during the study.

### 2.2. Procedure

The study was programmed with lab.js [38] and conducted online. After agreeing to the experimental terms, the first session of the *mind game* commenced (experimental instructions for the whole experiment are available in the electronic supplementary material). Participants had to come up with a random number between 1 and 8, which they should write on a piece of paper. Only after they had chosen their number they should proceed to the next screen, on which a random number from this range was shown. We asked participants to indicate via keypress whether their chosen number was the same as the one presented on the screen (*J* = ‘yes’, *F* = ‘no’). We explained that coming up with the same number as the one on the screen would lead to an additional bonus of £0.50. Due to the online setup, the experimenter had obviously no opportunity to check the veracity of this response or to access the identity of any participant. Thus, there was a financial incentive for unethical behaviour (i.e. lying), with no chances of getting caught or being punished.

After indicating their response in the mind game, the study proceeded with the thinking-style manipulation. Participants in the divergent thinking group were asked to come up with as many uses for two given objects as possible (pen and bottle; *alternative uses task* [32]). The two objects were presented one after the other, with 5 min for responding per item. After the responding phase of each item, we presented suitable exemplary responses for 15 s. We decided to provide such feedback to create similar feedback experiences in all three tasks. Participants in the convergent thinking group worked on the *remote associates task* [33]. In each trial, they saw three terms (e.g. fish, mine, rush) for which they had to find a common associate (here: gold). There were 15 trials, each lasting 40 s, and the correct response was displayed for 2 s. By contrast, participants in the rule-based group had to classify two visual stimuli (i.e. ‘mechanic’ or ‘organic’) depending on a simple rule (if you see the label for mechanic, please press *S*/*L*; if you see the label for organic please press *L*/*S*). Stimulus–response mappings were determined randomly for each participant, but they were constant across the entire task. The stimulus was presented for 2 s, preceded by the presentation of a fixation cross for also 2 s. To ensure that each trial took the same amount of time, feedback was shown for 1 s plus the difference of the reaction time of the respective trial and 2 s. There were two blocks with 53 trials each and there were breaks of 20 s before each block and, additionally, of 30 s after each block.

In total, the thinking-style manipulation took 10.5 min in each group. Then the second session of the mind game followed, with the same procedure as for the first run. Afterwards, participants had to indicate how much they feel constrained by rules on a visual slider going from ‘Not at all’ to ‘Very much’. Responses were scaled from 0 to 100. At the very end of the experiment, we included the *divergent association task* (DAT) as a measure of verbal creativity [39]. Participants had 4 min to come up with ten words as different from each other as possible, regarding all uses and meanings. We included this task, to test whether trait creativity (measured via the DAT) is positively linked to unethical behaviour, only if this trait is activated by a corresponding creativity task, as suggested by previous research [40]. To be precise, such reasoning would imply a positive correlation of the DAT score and the behaviour in the mind game, only for the divergent and the convergent thinking group, but not for the control group. Furthermore, we employed these two final tasks to explore a potential link of creativity and unethical behaviour (via the extent to which one feels constrained by rules) and to analyse whether such a potential link is moderated by dispositional creativity.

### 2.3. Data treatment and analyses

Before conducting our analyses, we excluded datasets of participants with low performance in the thinking style tasks. For the divergent thinking group, datasets with on average less than three valid uses per object in the *alternative uses task* were excluded. For the convergent thinking group, participants had to solve at least three questions in the *remote associates task* correctly to be included in the analyses and for the rule-based group, participants’ share of correct responses in the classification task had to be 75% or higher.

We calculated a logistic regression with participants' responses in the mind game (same number as the one presented on the screen or not) as dependent variable, using mind game time-point (before versus after the thinking style manipulation) and thinking style (divergent versus convergent versus rule-based) as well as the interaction of the two factors as predictors (all three predictors entered the model in a single step). We also included contrasts in this analysis that addressed our hypotheses and that controlled for participant responses before the thinking style manipulation. For one, we employed an indicator contrast for the factor mind game timing with the time-point before the thinking style manipulation as a reference (i.e. coding of the contrast: 0, 1). Second, we employed a difference contrast for the thinking style variable. This contrast allowed a comparison of the convergent to the divergent thinking group (i.e. coding of the contrast: −0.500, + 0.500, 0.000) and a comparison of the rule-based group to the mean of the convergent and the divergent thinking group (i.e. coding of the contrast: −0.333, −0.333, 0.667).

We also conducted several exploratory analyses. To test whether the DAT score, which refers to the transformed average of the semantic distances between the first seven valid responses out of the ten entered words, differed between the thinking style groups, we computed an analysis of variance (ANOVA) with the between-subjects factor thinking style. In the case of a significant main effect, we compared the DAT scores between each pair of thinking styles in two-tailed two sample *t*-tests. Whether the DAT score moderated the influence of thinking style on dishonesty was tested in another logistic regression with participants’ responses in the mind game as dependent variable. In addition to mind game timing, thinking style and the interaction of both factors, we used the DAT score as well as the interaction of mind game timing, thinking style and DAT score as predictors (all predictors entered the model in a single step). To analyse whether a potential effect of thinking style on dishonesty is due to feelings of being less constrained by rules, we compared ratings of rule-constrainedness between thinking style groups with the same statistical approach as described above for the DAT score. Further, we tested whether this rating moderates the influence of thinking style on dishonesty, also following the same statistical approach as described above for the DAT score. Finally, we included exploratory analyses that we did not preregister: We calculated the correlation of the DAT score and the number of times participants indicated that they noted down the same number as presented on the screen (i.e. both numbers different, one number identical or both numbers identical). We performed this analysis for the whole sample as well as separately for each thinking style group.

## 3. Results

Raw data, analysis syntax and program file are available on the Open Science Framework (https://osf.io/4qxu8) [41]. Performance in the thinking style tasks was comparable to earlier studies, using similar manipulations to induce convergent and divergent thinking (e.g. [29]). Participants solved on average 8 out of 15 (s.d. = 3.23) questions in the remote associates task correctly (convergent thinking), came up with on average 8 (s.d. = 3.81) possible uses per object (divergent thinking), and correctly classified about 103 out of 106 (s.d. = 2.84) of all labels in the classification task (rule-based thinking). Note that these values apply to the final sample (i.e. after data exclusions as described above).

The logistic regression model was statistically significant (*X^2^* (5, *N* = 450) = 50.22, *p <* 0.001). The model explained 7.4% (Nagelkerke *R*^2^) of the variance in participants’ responses (same or different number as the one presented on the screen) and correctly classified 63.7% of cases. Table 1 presents results from the logistic regression.

**Table 1.** Results from the logistic regression of reporting the same number as indicated on the screen within the mind game. *Thinking style*: convergent versus divergent versus rule-based thinking. *Time-point*: before versus after the thinking style manipulation. Numbers in parentheses indicate specific contrasts, i.e. a difference contrast for the thinking style (1 = convergent versus divergent, 2 = averaged convergent and divergent versus rule-based) and an indicator contrast for the time-point (the time-point before the thinking style manipulation serves as the reference). Note that the contrasts of the thinking style predictor test for differences at the reference time-point only. Standard errors in parentheses. ***p < 0.001, **p < 0.01, *p < 0.05.

variable	*β*
thinking style	
thinking style (1)	−0.41 (0.26)
thinking style (2)	−0.09 (0.23)
time-point (1)	0.95*** (0.14)
thinking style × time-point	
thinking style (1) by time-point (1)	0.14 (0.35)
thinking style (2) by time-point (1)	−0.12 (0.30)
constant	−1.02*** (0.11)
*N*	450

**Figure 1.**
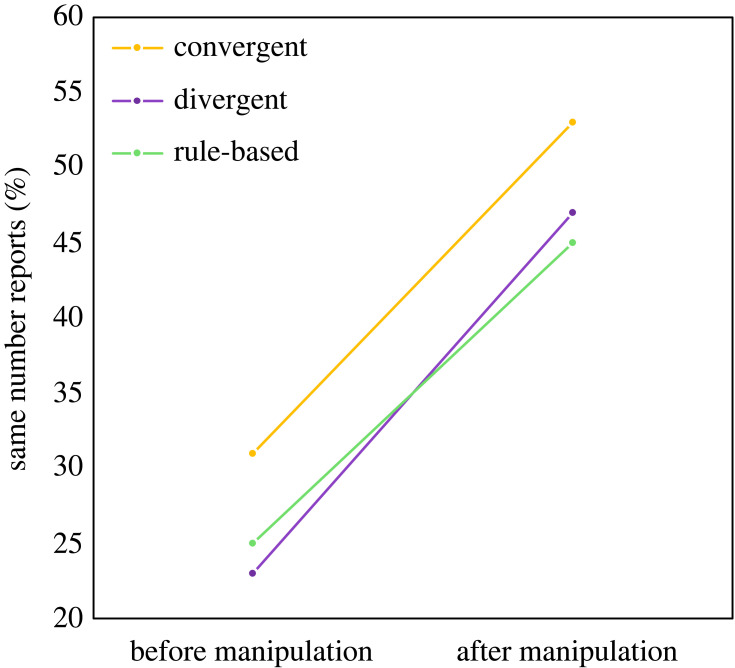
Share of participants who indicated the same number as the one presented on the screen, before and after the thinking style manipulation. The colours of the lines represent the different thinking style groups (convergent thinking: yellow, divergent thinking: purple, rule-based thinking: green). The chance level of coming up with the same number as presented on screen is 12.5%.

Controlling for the time-point before the thinking style manipulation, the probability of a same number report did not differ between thinking style groups (*Wald* $\chi_{2}^{2}=2.65$, *p* = 0.266). However, more same numbers were reported after than before the thinking style manipulation (*Wald* $\chi_{1}^{2}=43.85$, *p* < 0.001, OR = 2.58, 95% CI = [1.95, 3.41]). Importantly, we neither found an interaction between time and thinking style group for the comparison of the divergent and the convergent thinking group, nor for the comparison of the average of both creative thinking groups and the rule-based thinking group (figure 1; *Wald* $\chi_{s}^{2}<1$).

DAT scores did not differ between thinking style groups (convergent thinking: *M* = 76.60, s.d. = 5.66; divergent thinking: *M* = 76.07, s.d. = 6.14; rule-based thinking: *M* = 76.89, s.d. = 5.41; *F* < 1). Note that for 8% (*n* = 36) of the participants, no DAT score could be calculated as these individuals entered fewer than seven valid responses. The logistic regression model reached significance (*X^2^* (8, *N* = 414) = 45.58, *p <* 0.001) and explained 7.3% (Nagelkerke *R*^2^) of the variance in participants’ *mind game* responses with 63.2% of cases being correctly classified. Table 2 presents results from the logistic regression.

**Table 2.** Results from the logistic regression of reporting the same number as indicated on the screen within the mind game, additionally including the DAT score as a predictor. *Thinking style*: convergent versus divergent versus rule-based thinking. *Time-point*: before versus after the thinking style manipulation. *DAT*: score in the divergent association task. Numbers in parentheses indicate specific contrasts, i.e. a difference contrast for the thinking style (1 = convergent versus divergent, 2 = averaged convergent and divergent versus rule-based) and an indicator contrast for the time-point (the time-point before the thinking style manipulation serves as the reference). Note that the contrasts of the thinking style predictor test for differences at the reference time-point only. Standard errors in parentheses. ***p < 0.001, **p < 0.01, *p < 0.05.

variable	*β*
thinking style	
thinking style (1)	−0.40 (0.27)
thinking style (2)	−0.07 (0.24)
time-point (1)	0.90*** (0.15)
thinking style × time-point	
thinking style (1) by time-point (1)	4.73 (3.18)
thinking style (2) by time-point (1)	2.01 (2.96)
DAT	0.01 (0.01)
time-point × thinking style × DAT	
time-point (1) by thinking style (1) by DAT	−0.06 (0.04)
time-point (1) by thinking style (2) by DAT	−0.03 (0.04)
constant	−1.71 (1.01)
*N*	414

The results on thinking style, time-point and the interaction of the two predictors mirrored the preceding analyses. Importantly, neither the DAT score nor the interaction of *mind game* timing, thinking style group and DAT score predicted participants' responses in the *mind game* (*Wald* $\chi_{s}^{2}\leq 2.10$, *p* ≥ 0.147).

Further, ratings for feelings of being constrained by rules did not differ between thinking style groups (convergent thinking: *M* = 45.77, s.d. = 28.32; divergent thinking: *M* = 43.74, s.d. = 30.34; rule-based thinking: *M* = 42.39, s.d. = 32.04; *F* < 1). The logistic regression model was significant (*X^2^* (8, *N* = 450) = 53.18, *p <* 0.001) and explained 7.8% (Nagelkerke *R*^2^) of the variance in participants’ *mind game* responses. In this model, 63.3% of cases were correctly classified. Table 3 presents results from the logistic regression.

**Table 3.** Results from the logistic regression of reporting the same number as indicated on the screen within the mind game, additionally including ratings of rule-constrainedness as a predictor. *Thinking style*: convergent versus divergent versus rule-based thinking. *Time-point*: before versus after the thinking style manipulation. *Rule-constrainedness*: rating of rule-constrainedness. Numbers in parentheses indicate specific contrasts, i.e. a difference contrast for the thinking style (1 = convergent versus divergent, 2 = averaged convergent and divergent versus rule-based) and an indicator contrast for the time-point (the time-point before the thinking style manipulation serves as the reference). Note that the contrasts of the thinking style predictor test for differences at the reference time-point only. Standard errors in parentheses. ***p < 0.001, **p < 0.01, *p < 0.05.

variable	*β*
thinking style	
thinking style (1)	−0.41 (0.26)
thinking style (2)	−0.10 (0.23)
time-point (1)	0.95*** (0.14)
thinking style × time-point	
thinking style (1) by time-point (1)	0.12 (0.50)
thinking style (2) by time-point (1)	−0.33 (0.41)
rule-constrainedness	<|0.01| (<|0.01|)
time-point × thinking style × rule-constrainedness	
time-point (1) by thinking style (1) by rule-constrainedness	<|0.01| (0.01)
time-point (1) by thinking style (2) by rule-constrainedness	0.01 (0.01)
constant	−0.86*** (0.15)
* N*	450

The results on thinking style, time-point and the interaction of the two predictors were again in line with our main analyses. Crucially, neither rule-constrainedness ratings (*Wald* $\chi_{1}^{2}=2.52$, *p* = 0.113, OR = 1.00, 95% CI = [0.99, 1.00]), nor the interaction of *mind game* timing, thinking style group and rule-constrainedness ratings predicted participants’ responses in the *mind game* (*Wald* $\chi_{s}^{2}<1$).

Finally, neither the whole sample, nor any particular thinking style group showed a relation of the DAT score and the number of times participants indicated that they had noted down the same number as presented on the screen (|*r|*s ≤ 0.09, *t*s ≤ 1.11, *p*s ≥ 0.271).

## 4. Discussion

Does creativity increase dishonesty? We studied this question by manipulating the mindsets of our participants via different tasks. Therefore, we had two creative thinking groups (divergent and convergent thinking) as well as a reference group conducting a simple classification task (rule-based thinking). Before and after these tasks, participants conducted the *mind game*, as an unobtrusive and straightforward measure of dishonesty. Even though we found a remarkable increase of dishonesty from the first to the second time participants conducted the mind game, there was no credible evidence that this development was dependent on the induced thinking style. Moreover, the influence of the creativity tasks on dishonesty was not moderated by dispositional creativity or feelings of being constrained by rules. Also, additional exploratory analyses did not support a relation of trait creativity and dishonesty.

These results contradict earlier findings of increased dishonesty for a creative compared to a traditional prime [18]. As our setup differs from previous studies in several ways, there are multiple potential reasons for this diverging outcome. Most importantly, we used different tasks to induce a creative mindset and to measure dishonesty. It has been argued that the influence of creativity on unethical behaviour is due to an enhanced ability to come up with self-serving, unethical justifications and thus, this link should be mainly present when it is difficult to justify dishonest behaviour [18,40]. Even though it is not clear whether the *mind game*, the *matrices task* [18,42] and the *die-under-the-cup paradigm* [18,43,44] differ in their difficulty of justifying dishonest behaviour, we judge these setups to be largely similar, so we do not assume that this methodological difference drives the divergence of results across studies. Moreover, as recently shown, the relation of cognitive flexibility and creativity is quite ambiguous [45] and creativity does not seem to be exclusively related to the generation of unethical justifications but to the production of justifications in general [46]. Speculatively, creative thinking might lead to more ethical justifications for some participants, thus promoting honesty, while other participants might come up with more unethical justifications, therefore opting for dishonest behaviour. These two contradictory influences may have cancelled each other out, resulting in no difference in comparison with the rule-based thinking group overall.

A more relevant limitation of our setup could be that our mindset induction tasks might not only differ in the extent they stimulate creative thinking but also in further characteristics like required cognitive capacity. As cognitive load has been found to promote honesty [47,48], such an effect might have counteracted a potential influence of the respective thinking style.

A final crucial difference from previous work is statistical power. The groups in the present study (*n* = 150 per group) were three to four times larger than those used in previous work (e.g. around *n* = 40 to *n* = 56 for Experiment 2–3 in [18]). Because smaller groups are conducive to false positive findings, the present results might indeed indicate that there is no direct impact of creativity on dishonest behaviour in general.

Overall, we observed a considerable level of dishonesty which, independent of the induced mindset, even increased substantially from the first to the second measurement time. This outcome supports the assumption that many people lie when it is rewarding and there is no chance of getting caught or punished [43]. Due to the online setup, perceived anonymity might have been particularly high in the present experiment, therefore leading to a substantial share of dishonest responses (see also [49]). Furthermore, our results corroborate earlier findings of increased dishonesty over time (e.g. [50]). This surge of lying behaviour might be due to a habituation to the negative affective response induced by dishonest behaviour [51] (but see [47] for an opposing perspective) or due to an increased awareness about the opportunity to lie from the second compared to the first run of the *mind game* [52]. Finally, participants might have had the feeling that we implicitly encouraged them to choose the financially more rewarding option and such demand effects might have been particularly pronounced at the second time participants encountered the mind game (see also [53]).

Overall, our results are in line with those of earlier correlational studies, suggesting that the relation of creativity and unethical behaviour is not a simple one and depends on several contextual factors. For instance, prior research found a positive relation of subjective creativity and dishonest responses in the mind game, while the opposite relation was found for objective creativity [26]. Moreover, is has been shown that trait creativity is only linked to deviant behaviour in organizational settings for individuals low in moral identity [54] and another study only found a relation of dishonesty and trait creativity when participants were put in a creative state beforehand [40]. Such findings highlight that creativity is a broad construct with different facets [55] which is linked to various personality traits depending on the detailed context [56]. This heterogeneity within the creative spectrum also leads to methodological challenges regarding its measurement [57]. In the present study, we found no relation of trait creativity, measured via the DAT, and dishonesty in the *mind game*, irrespective if participants were in a creative state or not. As the DAT focuses on divergent thinking skills within the verbal domain, different results might have been obtained for trait measures of other creativity facets like convergent thinking.

## 5. Conclusion

Our results indicate that the relation of creativity and unethical behaviour seems to be even more ambiguous than previously assumed, not only regarding trait creativity but also regarding short-term priming of a creative thinking style. However, addressing this issue would be of utmost importance for various practical settings like educational facilities or workplace environments, where promoting creative ideas is usually a key goal [10,58]. Whether or not interventions which aim to enhance creative thinking (e.g. [59]) come with negative side effects like increased dishonesty is clearly highly relevant for decision-makers in these settings. Importantly, our results do not support such a pessimistic view, at least not for the majority of human agents. At the same time, there is still a lot of room for future research, addressing potential moderators of this relationship like further personality traits and situational characteristics.

## Data Availability

Raw data, analysis syntax and program file are available on the Open Science Framework (https://osf.io/4qxu8). Supplementary material is available online [60].

## Footnotes

1 Note that despite this widespread assumption, recent meta-analyses indicate only a small, yet positive, correlation of intelligence and divergent thinking (*r* = 0.25 in [8]). Similar results were found regarding the relation of intelligence and creative achievements (*r* = 0.16 in [9]).

2 Note that regression results do not differ substantially when instead using datasets of the first 150 participants per group (independent of performance in the inducer task). This renders it highly implausible that our results were caused by reduced variance within the independent variable that might have come with our preregistered exclusion criteria. Corresponding regression tables are presented in electronic supplementary material, tables S1–S3.
